# Africa’s ownership of a sustainable health journey towards an AIDS-free future

**DOI:** 10.1136/bmjgh-2024-018198

**Published:** 2026-06-17

**Authors:** Mumbi Chola, Magda Robalo, Kent Buse, Pokuaa Oduro-Bonsrah, Jekwu Ozoemene, Abdoul Dieng, Ruth Akulu, Bernard Madzima, Awa Marie Coll-Seck, Robb Sheneberger, Sesupo Makakole Nene, Mark Dybul, Izukanji Sikazwe, Michel Sidibe, The African-led HIV Control Workng Group, Magda Robalo

**Affiliations:** 1 Center for Infectious Disease Research in Zambia, Lusaka, Zambia; 2 Institute for Global Health and Development (IGHD), Bissau, Guinea-Bissau; 3 Global Health 50/50, Cambridge, UK; 4 HIV Trust Fund of Nigeria, Lagos, Nigeria; 5 Nigeria Business Coalition Against AIDS (NiBUCAA), Lagos, Nigeria; 6 Consultant, Abidjan, Côte d’Ivoire; 7 ACTS101, Kampala, Uganda; 8 Zimbabwe National AIDS Council, Harare, Zimbabwe; 9 Galien Forum Africa, Dakar, Senegal; 10 Center for Infectious Disease Research in Zambia, Lusaka, Lusaka, Zambia; 11 SCMN Global Health Consulting, Pretoria, South Africa; 12 Center for Global Health Practice and Impact, Washington, Washington, USA; 13 Chief Executive Officer, Center for Infectious Disease Research in Zambia, Lusaka, Lusaka, Zambia; 14 Africa Medicines Agency, Bamako, Mali; 15 The African-led HIV Control Workng Group, Lusaka, Zambia

**Keywords:** Global Health, Health policy, Health systems, HIV, Africa

**Summary**

- Progress on Africa’s HIV response is under threat due to dependence on external models and underinvestment from African countries.
- Africa must redefine its health agenda and HIV response with locally designed and financed strategies, rooted in regional collaboration and solidarity.
- Africa must redefine its health agenda and HIV response with locally designed and financed strategies, rooted in regional collaboration and solidarity.

## Introduction

 Africa is on the brink of an evolutionary leap, transitioning from dependency on external aid to establishing itself as an autonomous and sustainable hub for innovation. Despite significant progress in the HIV response,^1^ over-reliance on imported models of service delivery and limited African investments threatens this journey. A shift towards self-reliance is both needed and feasible, as Africa possesses the minds and resources required.^2^ In view of urgent and diverse health challenges, Africa must redefine its health and HIV agenda, moving from one-size-fits-all solutions to tailored local strategies rooted in its diverse cultures and intracontinental collaboration. Funding-related barriers must be addressed, and international initiatives should align with Africa’s priorities.

The 12-member African-led HIV Control Working Group (HCWG) aims to redefine Africa’s health and HIV agenda by advocating for a sustainable, African-owned HIV response. The group advocates for a cooperative approach based on social justice, dignity, cultural diversity, health, transparency and community empowerment, decolonising global health architecture, while embracing the ethos of Ubuntu.

## Ensuring African-led governance, leadership and accountability

The current HIV governance structures are led by the global north, despite Africa bearing the largest HIV burden. African representation in decision-making bodies remains disproportionately low. Less than 5% of global health governance board seats are held by nationals from low-income countries (LMICs), of which only 1% of these are women.^3^ The HCWG is advocating for a governance model that addresses the asymmetries and amplifies African voices in global health decision-making through meaningful participation and representation. This will align global health leadership with local contexts and support the development of evidence-informed policies that address Africa’s specific priorities. Robust mutual accountability that serves African interests must be embedded within global governance systems to reset partnerships and place African priorities at the core of global HIV decision-making.

## Investing in our future: an African-owned Global Health and Wellbeing Fund

Over the past three decades, financial assistance for health, including HIV, has driven significant gains in LMICs. Yet, recent times have witnessed a slowdown in external finance, often aggravated by multiple crises such as COVID-19 pandemic and natural disasters, resulting in competing public health priorities and a reduction or flat-lining of funding needed for the HIV response,^4^ underscoring the urgent need for change. The group proposes an African-owned Global Health and Wellbeing Fund, embodying Africa’s commitment to control its health destiny, promoting empowerment and ownership. It is more than a financial tool; it asserts Africa’s broader security and autonomy over health resources, disrupting traditional aid models and aligning with the African Union’s Agenda 2063, ‘The Africa we want’. By leveraging strategic taxes, diaspora bonds, private sector engagement and the emerging philanthropic sector, the Fund aims to create a self-sustaining health financing model driven by African leadership and accountability.

## Shaping an African-led research and development infrastructure

Africa must lead the development of a continental-wide research and development (R&D) agenda that provides locally relevant solutions based on continental priorities to reshape its healthcare future. To realise this vision, communities must be included at every stage of the research process, ensuring that projects remain rooted in the realities of those they are meant to serve. Increasing funding for R&D from within Africa is crucial and should be supported by governments, the private sector and philanthropic organisations. Additionally, ensuring African sovereignty over research data and public health records is vital. Fostering mutual scientific accountability among researchers and leveraging South-South collaborations will further strengthen Africa’s capacity, while ensuring that South-North partnerships are more equitable and support African interests.

## Advancing medicines security and sovereignty through African-led innovation

Africa must prioritise local production of medicines, vaccines and other health products to ensure health security and drive economic growth.^5 6^ Transforming the continent’s healthcare system requires a shift towards local manufacturing^7^ and biotechnology systems, alongside cultivating a robust workforce of African scientists, technicians and healthcare professionals. This is pivotal in establishing health security and sovereignty, strengthening Africa’s health responses to ongoing and future health challenges. It demands harmonising regional and continental regulatory processes^6^ as well as creating unified markets and trade agreements. This approach ultimately improves access to equitable, quality healthcare by reducing inefficiencies and facilitating expedited approvals and implementation of activities.

## Building health systems with communities at the core

The HIV crisis requires communities at risk to be at the centre of health initiatives, empowering them to take control of their health outcomes and advocate for necessary services. This fosters trust, communication and partnerships between communities and healthcare providers, leading to more effective and sustainable interventions. Prioritising community engagement, including involvement in health governance, is not only about health equity but also a strategic imperative for building resilient health systems that can adapt to evolving challenges. Furthermore, as the youth population continues to grow, meaningful youth engagement is crucial in planning and decision-making processes for sustaining the HIV response.

## Realising health as a right

Recognising health as an inalienable right^8^ requires a multifaceted approach that actively includes HIV-affected people in the decision-making. This means transforming health, social and legal systems that prioritise individual and community health needs as a fundamental right, rather than privileges determined by socioeconomic status, gender, geography or other characteristics that put people at particular risk. Recognising the intersectional vulnerability of many people across any country creates deeper obligations for governments to act. This transformation necessitates comprehensive policy and legal reforms to ensure that marginalised communities are no longer excluded, that healthcare services are universal and that barriers to inclusion are dismantled. Shifting societal norms to view health as a basic human right requires prioritising investment in the public health infrastructure, including workforce recruitment, training and retention, particularly of community health workers, as well as health education for the general population that emphasises equity and accessibility. Additionally, implementing efficient financing mechanisms is essential to fairly distribute health costs and eliminate financial barriers. By embedding health as a right into legislation, policy, culture and practice, we can build more equitable, resilient and accountable systems capable of addressing future challenges.

## Integrating health systems and services

Integrating HIV services into the general healthcare infrastructure is imperative,^9^ especially for mitigating external shocks such as environmental disasters, political instability and civil unrest. This integration should extend beyond health services to encompass social, economic and technological factors, including the use of digital health technologies. Such a data-driven approach strengthens the overall resilience of the healthcare system and enables a more coordinated, effective response to a range of crises. Furthermore, integration enhances people-centred care and equitable healthcare delivery and optimises the use of resources, thus minimising bureaucratic inefficiencies and improving resource management practices. By adopting a holistic approach that incorporates HIV services into routine healthcare delivery, we can address the health needs of individuals comprehensively, streamline access to services and ensure continuity of care even in times of uncertainty. This approach can also help destigmatise HIV by providing ongoing support to affected individuals, fostering an inclusive health environment.

## Preparing and responding to the ‘Age of Pandemics’^10^

Outbreaks experienced on the continent during the last decade, such as COVID-19, Ebola^11^ (if by chronological order, Ebola should precede COVID-19), Cholera^12^ and Mpox,^13^ have intensified calls for robust pandemic prevention, preparedness and response mechanisms. These crises, along with ongoing conflicts and climate challenges, highlight the necessity of resilience planning that addresses multiple interconnected threats. Strong supply chains and sustainable financing strategies are crucial components of this planning. Insights from the HIV response and corresponding infrastructure developed over the years, such as prioritising community engagement, respecting human rights and providing holistic care, offer fundamental components for pandemic preparedness and response (PPR). Drawing insights from Africa’s responses to COVID-19 and HIV and leveraging established health and community systems will enhance global efforts in PPR.^14^

## Tailoring responses to African-specific health agendas

HIV responses must be tailored to address the diverse needs of specific populations and contexts, including adolescent girls and young women, key and vulnerable populations, and young people. Responses should also meaningfully include insights and practices of traditional and community healers and leaders. An African-owned AIDS agenda will improve our ability to pay greater attention to Africa’s diversity, cultures and norms; improve access to healthcare; and promote health and well-being for all.

## Conclusion

Africa must transition towards self-sustainability to secure the health of its citizens. With millions of Africans living with HIV and an expanding array of competing public health priorities, the continent must increasingly look inward for sustainable solutions and lead a transformative health agenda. The continent has all it takes to lead, design and deliver healthcare services that respond to the aspirations of its people and become an active player in the global health ecosystem.

## Data Availability

There are no data in this work.
